# Detection of Extended-spectrum β-lactamase-producing *Escherichia coli* isolates by isothermal amplification and association of their virulence genes and phylogroups with extraintestinal infection

**DOI:** 10.1038/s41598-023-39228-w

**Published:** 2023-07-25

**Authors:** Naeem Ullah, Thadchaporn Assawakongkarat, Yukihiro Akeda, Nuntaree Chaichanawongsaroj

**Affiliations:** 1 Research Unit of Innovative Diagnosis of Antimicrobial Resistance, Department of Transfusion Medicine and Clinical Microbiology, Faculty of Allied Health Sciences, Chulalongkorn University, Bangkok, Thailand; 2 Program of Molecular Sciences in Medical Microbiology and Immunology, Department of Transfusion Medicine and Clinical Microbiology, Faculty of Allied Health Sciences, Chulalongkorn University, Bangkok, Thailand; 3 Department of Bacteriology I, National Institute of Infectious Diseases (NIID), Tokyo, Japan

**Keywords:** Microbiology, Molecular biology

## Abstract

Extraintestinal pathogenic *Escherichia coli* (ExPEC) producing extended-spectrum β-lactamases (ESBL) cause serious human infections due to their virulence and multidrug resistance (MDR) profiles. We characterized 144 ExPEC strains (collected from a tertiary cancer institute) in terms of antimicrobial susceptibility spectrum, ESBL variants, virulence factors (VF) patterns, and Clermont’s phylogroup classification. The developed multiplex recombinase polymerase amplification and thermophilic helicase-dependent amplification (tHDA) assays for *bla*_CTX-M_, *bla*_OXA_, *bla*_SHV*,*_ and *bla*_TEM_ detection, respectively, were validated using PCR-sequencing results. All ESBL-ExPEC isolates carried *bla*_CTX-M_ genes with following prevalence frequency of variants: *bla*_CTX-M-15_ (50.5%) > *bla*_CTX-M-55_ (17.9%) > *bla*_CTX-M-27_ (16.8%) > *bla*_CTX-M-14_ (14.7%). The multiplex recombinase polymerase amplification assay had 100% sensitivity, and specificity for *bla*_CTX-M_, *bla*_OXA_, *bla*_SHV_, while tHDA had 86.89% sensitivity, and 100% specificity for *bla*_TEM_. The VF genes showed the following prevalence frequency: *tra*T (67.4%) > *omp*T (52.6%) > *iut*A (50.5%) > *fim*H (47.4%) > *iha* (33.7%) > *hly*A (26.3%) > *pap*C (12.6%) > *cva*C (3.2%), in ESBL-ExPEC isolates which belonged to phylogroups A (28.4%), B2 (28.4%), and F (22.1%). The distribution of *tra*T, *omp*T, and *hly*A and phylogroup B2 were significantly different (*P* < 0.05) between ESBL-ExPEC and non-ESBL-ExPEC isolates. Thus, these equipment-free isothermal resistance gene amplification assays contribute to effective treatment and control of virulent ExPEC, especially antimicrobial resistance strains.

## Introduction

Extended-spectrum β-lactamases (ESBLs) in *Enterobacteriaceae* are categorized by World Health Organization as the most critical cause of antimicrobial resistance (AMR) requiring discovery of new antibiotics^1^. In addition, most ESBL-producing *Enterobacteriaceae* are also multidrug-resistant (MDR), burdening the treatment^2^. Extraintestinal pathogenic *Escherichia coli* (ExPEC) is a major ESBL-producing organism which in addition to intestine, infects, urinary tract, bloodstream, meningitis, and wounds and causes sepsis. ESBL associated AMR in ExPEC is not only disseminated in health care settings but also in community-acquired infections^3^. The global increase of ESBL-ExPEC strains is causing clinical and economic losses similar in magnitude to that caused by pathogenic *E. coli*. Unlike for, intestinal pathogenic *E. coli* or commensal *E. coli*, defining the origin or primary reservoir of ExPEC is the major challenge in its treatment^4^. Moreover, the influence of AMR and virulence factors (VF) genes on ExPEC pathogenicity has become a serious global concern. Thus, researchers majorly rely on ExPEC genotyping to explore the association between AMR genes, VF, and their phylogenetic distribution.

The distribution of ESBL-producing *E. coli* (ESBL-*E coli*) in extraintestinal infections is diverse and varies among different geographical regions. The clonal spread of *E. coli* ST131 (associated with ExPEC infections, particularly urinary tract and blood stream infections) contributed to globally disseminated MDR clone^5^. Among the ESBL genes, *bla*_CTX-M-15_ is highly prevalent, followed by CTX-M, TEM, SHV, PER, VEB, GES, BES, TLA, and OXA genes^6^. The commensal *E. coli* in healthy cattle, pigs, and chickens serve as a reservoir of AMR genes^7^. The prevalence of CTX-M genes is higher in uropathogenic *E. coli* (UPEC) than in the commensal isolates from healthy volunteers^8^. Moreover, ESBLs producing *Enterobacterales* (ESBL-*Enterobacterales*) can colonize long-term (> 12 months) as intestinal microbiota^9^ enhancing the spread of ESBL AMR in a given health system, including humans, animals, and environments^10^. The widespread presence of ESBL genes in systemic infections also significantly impacts therapeutic and mortality outcomes. Clinical and Laboratory Standard Institute (CLSI) recommends phenotypic screening and confirmatory testing for ESBLs production as a part of regular clinical treatment of microbial infections^11^. However, the genotypic methods of ESBL screening are more advantageous for epidemiological management and help in overcoming challenges associated with phenotypic expression variance^12^.

According to Ambler’s molecular classification, ESBLs are serine β-lactamases comprising three significant class A genes (*bla*_CTX-M_, *bla*_TEM_, and *bla*_SHV_), and a class D *bla*_OXA_ gene. Recently, various newly discovered isothermal nucleic acid amplification techniques, which do not require expensive thermal cycling machines, have been extensively applied for rapid and simple detection of AMR genes^13^. In 2021, a 10 min naked-eye rapid multiplex recombinase polymerase amplification (RPA) lateral flow assay was developed to detect the three common ESBLs genes (*bla*_CTX-M_, *bla*_OXA_, and *bla*_SHV_)^14^. However, the use of engineered recombinase enzyme from *E. coli* K12 or BL21 strains (carrying their own *bla*_TEM_ gene) proved to be a major setback; the use of these RPA kits was plagued with cross reactivity and false positive results^15^. Helicase-dependent amplification technique, like RPA, utilizes a primer pair to amplify a specific DNA/RNA target at a set temperature. To unwind double-stranded DNA and generate a new amplicon, the RPA kit (TwistDx) uses recombinase and DNA polymerase with strand displacement activity (*Sau* polymerase), whereas the IsoAmp® II Universal tHDA kit (New England Biolabs) employs a thermostable helicase Tte-UvrD from *Thermoanaerobacter tengcongenesis* and *Bacillus stearothermophilus* DNA polymerase (*Bst*-DNA polymerase)^16^. The optimum amplification conditions for RPA and tHDA are 37–42 °C for 20–40 min and 60–65 °C for 90–120 min, respectively^17^. Both, RPA and tHDA, are portable nucleic acid detection assays ideally suited for use in point-of-care or resource-limited field settings^13^.

The ExPEC pathogenicity is influenced by several VF genes, with wide functions, ranging from bacterial colonization to virulence^18^. Moreover, most VF genes are encoded by plasmids, pathogenicity islands (PAI)^18,19^ or other mobile genetic elements^18^ which horizontally transfer to other pathogenic and non-pathogenic bacteria. *E. coli* is classified into eight phylogenetic groups (A, B1, B2, C, D, E, F, and Clade I) using the easier quadruplex PCR (which is less complex and more rapid than multilocus sequence typing or ribotyping methods)^20^. The association of ESBLs, VF genes, and phylogroups has been investigated in both ExPEC and commensal *E. coli*. Milenkov et al., found that *bla*_CTX-M-15_ was the most prevalent ESBL gene in *E. coli* specimens isolated from healthy pregnant Madagascar woman. In addition, 90% of these isolates belonged to phylogenetic groups A, B1, and C (which are associated with commensalism and carry a few VF genes involved in adhesion and iron acquisition). While, 10% of these isolates belonged to the extraintestinal virulent phylogenetic groups B2, D, and F^21^. Persistent long-term carriage (average 3.5 months) of ESBL-*Enterobacterales* (which belonged to extraintestinal virulence associated phylogroup B2/D/F) was observed in travelers visiting tropical areas, 3 or more months after their return. Whereas, commensal associated phylogroups A/B1/E persisted for shorter carriage durations (approx. 0.5 months)^21^. Phylogroup B2 of uropathogenic *E. coli* (UPEC) exhibited maximum AMR and carried six VFs^22^. ESBL-ExPECs carrying VF genes, isolated from bloodstream infections, showed the following order of phylogroup predominance: B2 (45.8%) > B1 (18.8%) > E (14.6%). The ESBL *bla*_CTX-M-15_ and VF *tra*T genes were the most predominant^23^. *E. coli*, the most frequent pathogen, second only to group B streptococci, causing neonatal meningitis in early-onset infections, belonged to extraintestinal phylogroup B2; > 70% of this pathogenic *E. coli* strain carry *kps*II, K1, *neu*C, *iuc*C, *sit*A, and *vat* genes. In contrast, *E. coli* obtained from healthy individuals belonged to groups A and D; they carry < 27% of VF genes^24,25^.

We aimed to develop isothermal amplification assays to characterize ESBL genes (*bla*_CTX-M_, *bla*_OXA_, *bla*_SHV_, and *bla*_TEM_) in ESBL-ExPEC strains. The sensitivity and specificity of these assays were validated using nucleotide sequencing. These simple isothermal platforms can be established in low resources settings for monitoring ESBL in all clinical samples. Moreover, we characterized and phylogenetically analyzed VF genes (*tra*T*, omp*T*, iut*A*, fim*H*, ih*a*, hly*A*, pap*C, and *cva*C) found in the ESBL-ExPEC clinical isolates. Identifying associations between ESBL genes, VF genes, and ESBL-ExPEC phylogroups is important for improved global AMR surveillance, targeted antibiotic treatment, and infection control.

## Results

### Identification of ESBLs, their variants, and antimicrobial susceptibility pattern

Of the 144 ExPEC isolated from various extraintestinal specimens including blood, urine, pus, body fluid, and sputum, 95 ESBL- and 49 non-ESBL-ExPEC strains were identified by combination disk method given in CLSI guideline^11^. All 95 ESBL-producing *E. coli* were subjected to genotyping. We found *bla*_CTX-M_ genes (n = 95) in single or in combination with *bla*_TEM_ (n = 40), *bla*_OXA_ (n = 29), and *bla*_SHV_ (n = 2) genes **(**Supplementary Table S1). In addition, 21 out of 49 phenotypically screened non-ESBL-producing *E. coli* harbored *bla*_TEM-1_ gene, while none of *bla*_TEM_, *bla*_OXA_, *bla*_SHV_, and *bla*_TEM_ genes were observed in the rest. The PCR-sequencing results of CTX-M variants revealed the following order of prevalence: *bla*_CTX-M-15_ (50.5%) > *bla*_CTX-M-55_ (17.9%) > *bla*_CTX-M-27_ (16.8%) > *bla*_CTX-M-14_ (14.7%). The *bla*_SHV_ was found only in 2 isolates. The antibiotic susceptibility pattern of most common ESBL–ExPEC variants (CTX-M-15, CTX-M-27, CTX-M-14, and CTX-M-55 types) showed 100% resistance to cefotaxime and cefdinir as described in Table 1. The following order (high–low) was observed in MDR toward ceftriaxone, cefepime, ciprofloxacin, ceftazidime, levofloxacin, and gentamycin: *bla*_CTX-M-15_ isolates > *bla*_CTX-M-55_ > *bla*_CTX-M-27_ isolates.

**Table 1:** The antimicrobial susceptibility pattern of *bla*_CTX-M_ variants in 95 ESBLs producing *E. coli.*

Antimicrobial drug group	Antimicrobial drug	Percentage			
		*bla*_CTX-M-14_ (N = 14)	*bla*_CTX-M-15_ (N = 48)	*bla*_CTX-M-27_ (N = 16)	*bla*_CTX-M-55_ (N = 17)
Penicillins	Ampicillin	100	97.9	94.1	100
Cephalosporins	Ceftazidime	7.1	72.9	41.2	87.5
	Cefotaxime	100	100	100	100
	Cefdinir	100	100	100	100
	Ceftriaxone	85.7	97.9	94.1	100
	Cefepime	57.1	91.7	41.2	93.8
Carbapenems	Doripenem	0	0	0	0
	Ertapenem	0	0	0	0
	Imipenem	0	0	0	0
	Meropenem	0	0	0	0
Fluoroquinolones	Ciprofloxacin	78.6	93.8	94.1	68.8
	Levofloxacin	71.4	93.8	94.1	68.8
Aminoglycoside	Amikacin	0	2.1	0	0
	Gentamicin	35.7	68.8	29.4	75
Combination drugs	Amoxicillin/Clavulanic acid	50	54.2	17.6	37.5
	Piperacilin/tazobactam	7.1	4.2	0	0

### Development of multiplex RPA and tHDA assays

**Figure 1:**
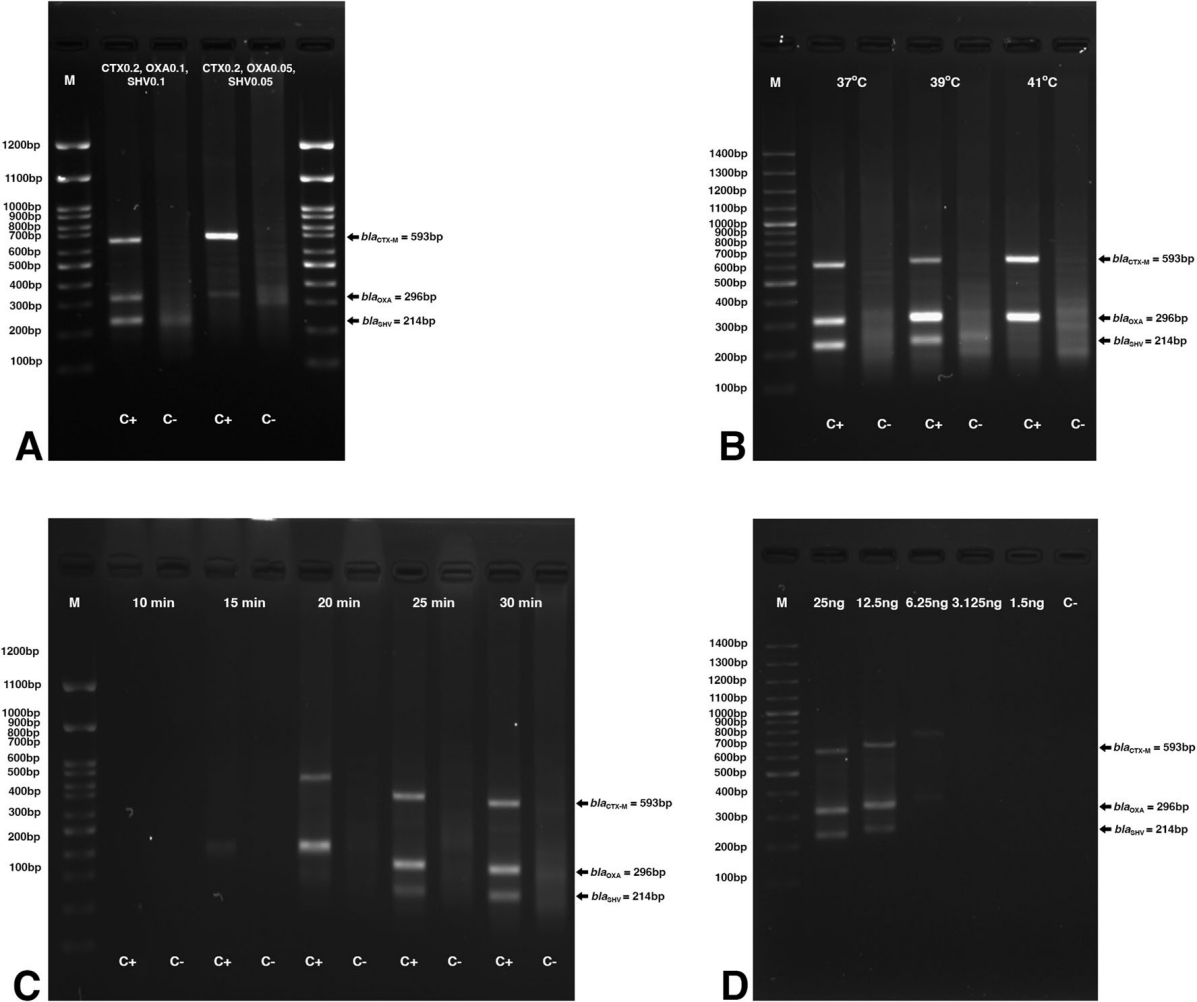
Optimization of the RPA assay. Agarose gel electrophoresis shows the *bla*_CTX-M_, *bla*_OXA_, and *bla*_SHV_ amplicons (**A**) when 0.2 µM CTX-M, 0.1 µM OXA, 0.1 µM SHV and 0.2 µM CTX-M, 0.05 µM OXA and 0.05 µM SHV were used; (**B**) at 37–41 °C; (**C**) at 10–30 min incubation; and (**D**) at different DNA template concentrations (1.5–25 ng) [M, 100 bp Marker; C+, positive DNA control; C−, no template control].

The optimum concentration of *bla*_CTX-M_, *bla*_OXA_, and *bla*_SHV_ primers required in multiplex RPA reaction were 0.2 µM CTX-M, 0.1 µM OXA and 0.1 µM SHV (Fig. 1A), respectively. All products were amplified at 37–39 °C (Fig. 1B) at 25–30 min (Fig. 1C). The DNA template concentration ranging at 12.5–25 ng showed the apparent bands of all 3 amplicons (Fig. 1D). The *bla*_SHV_ amplicon was absent at 41℃ and incubation duration < 25 min. Finally, the following multiplex RPA reaction condition was chosen as optimum: 37 °C for 25 min using 25 ng template DNA.

**Figure 2:**
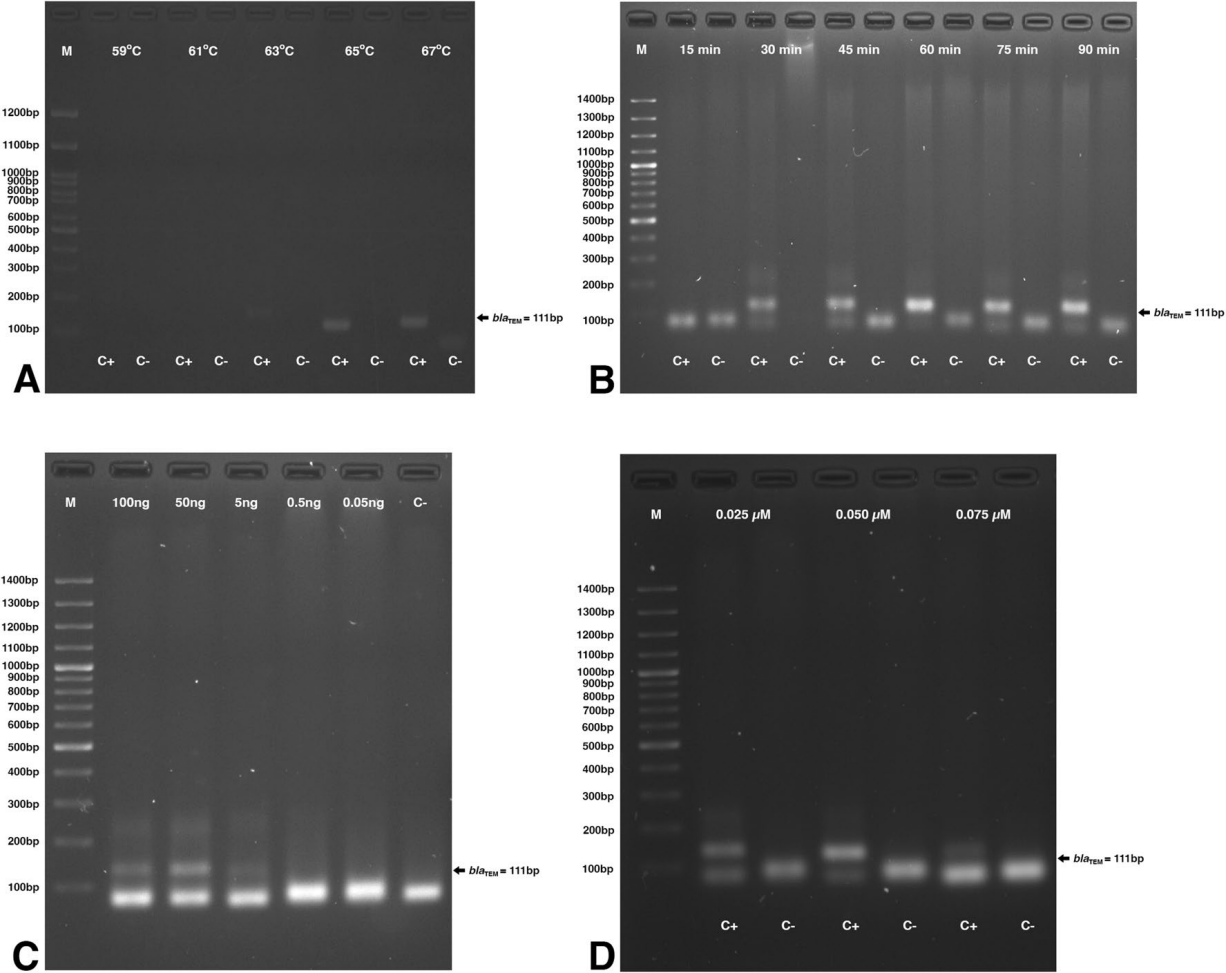
Optimization of the tHDA assay. Agarose gel electrophoresis showed the *bla*_TEM_ amplicons (**A**) at 59–67 °C; (**B**) at 15–90 min; (**C**) at different DNA template concentrations (0.05–100 ng); and (**D**) at different primers concentrations (0.025–0.075 µM) [M, 100 bp Marker; C+, positive DNA control; C−, no template control].

The tHDA was employed instead of RPA to amplify 111 bp of *bla*_TEM_. The *bla*_TEM_ amplicon was observed at 65–67 °C (Fig. 2A), with 30–90 min incubation (Fig. 2B). The optimum DNA template and primers concentrations were 50–100 ng (Fig. 2C) and 0.025–0.05 µM, respectively (Fig. 2D). The following tHDA condition was selected as optimum: 65 °C for 30 min using 50 ng template DNA and 0.025 µM primers.

### Limit of detection (LOD) and specificity of multiplex RPA and tHDA assays

**Figure 3:**
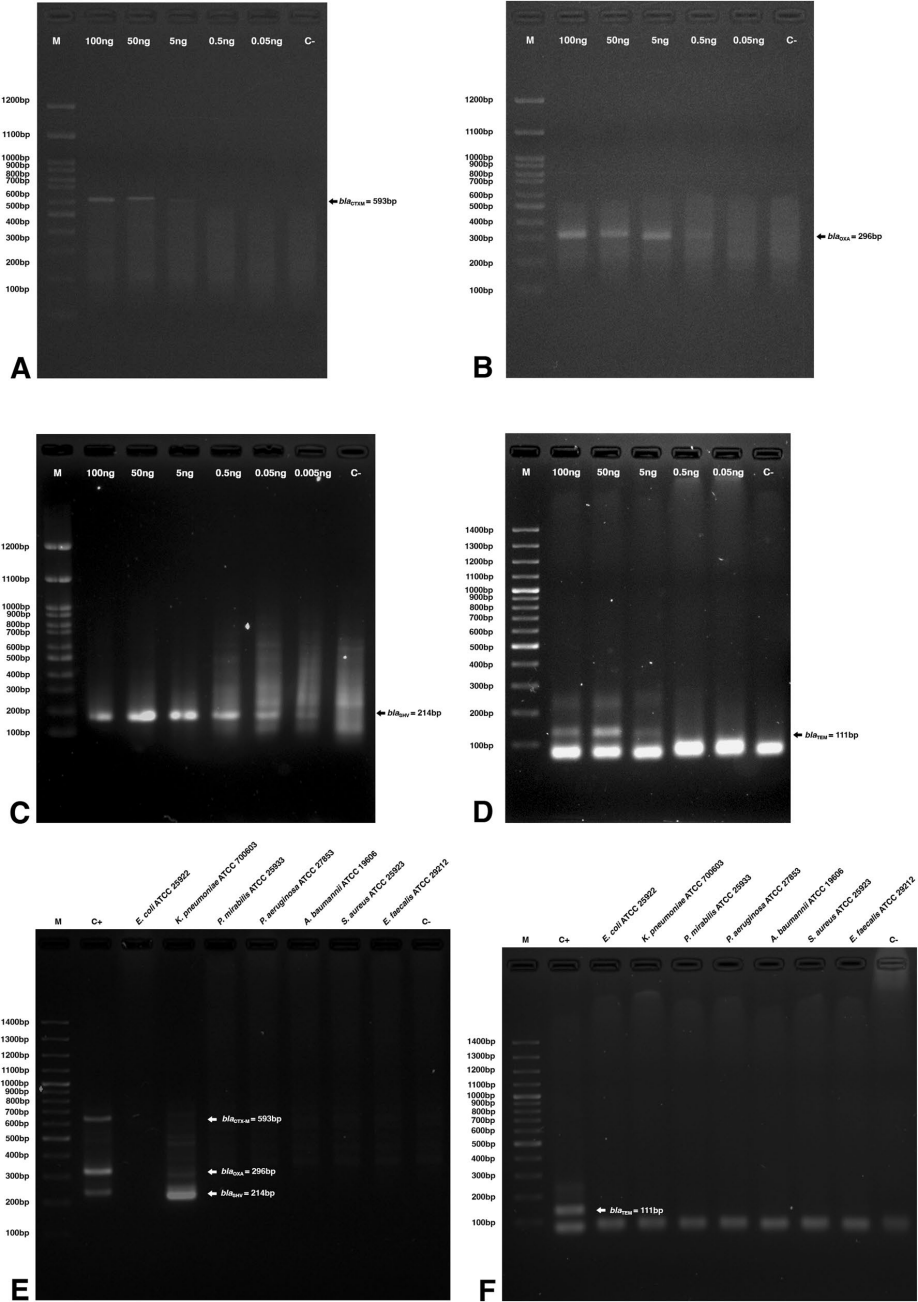
(**A–C**) LODs for *bla*_CTX-M_, *bla*_OXA_, and *bla*_SHV_ detection by RPA assay; (**D**) LODs for *bla*_TEM_ detection by tHDA; (**E**) Specificity of the RPA assay for *bla*_CTX-M_, *bla*_OXA_, and *bla*_SHV_ genes; and (**F**) Specificity of the tHDA assay for *bla*_TEM_ gene [M, 100 bp Marker; C+, positive DNA control; C−, no template control].

The DNA templates were isolated from *E. coli* clinical strain ESBL120 carrying *bla*_CTX-M_, strain KP125 carrying *bla*_OXA_, strain EC137 carrying *bla*_TEM_, and *K. pneumoniae* ATCC 700,603 carrying *bla*_SHV_. The LOD of multiplex RPA assays for *bla*_CTX-M_, *bla*_OXA_, *bla*_SHV_ genes (Fig. 3A–C) was 5 ng, 0.5 ng, and 0.5 ng, respectively. The sensitivity was 10–100 times higher than that of tHDA assay for *bla*_TEM_ (LOD of 50 ng) (Fig. 3D).

Amplicons were absent (Fig. 3E and F) in RPA and tHDA assays of the DNA of the following organisms: *E. coli* ATCC 25,922, *Proteus mirabilis* ATCC 25,933, *Pseudomonas aeruginosa* ATCC 27,853, *Acinetobacter baumannii* ATCC 19,606, *Staphylococcus aureus* ATCC 25,923 and *Enterococcus faecalis* ATCC 29,212. A 214 bp amplicon was produced from DNA of *K. pneumoniae* ATCC 700,603, which naturally contains *bla*_SHV_.

### Validation of multiplex RPA and tHDA assays

A total of 95 ESBL-ExPEC and 49 non-ESBL-ExPEC were subjected to multiplex RPA and tHDA assays. The results of *bla*_CTX-M_, *bla*_OXA_, and *bla*_SHV_ genes from multiplex RPA assays were in concordance with PCR-sequencing results exhibiting 100% sensitivity (95% CI = 96.19–100%, 88.06–100%, 15.81–100%, respectively), 100% specificity, positive predictive value (PPV), and negative predictive value (NPV) (95% CI: 92.75–100%, 96.84–100%, and 97.44–100%, respectively) (Table 2). While tHDA assay showed eight false-negative samples of *bla*_TEM_ gene yielding 86.89% (95% CI = 93.28–100), 100% (95% CI = 83.41–96.13%), 100% (95% CI = 28.93–45.42%), and 91.21% (95% CI = 77.37–92.78%) sensitivity, specificity, PPV, and NPV, respectively. The identification accuracy for *bla*_CTX-M_, *bla*_OXA_, and *bla*_SHV_ genes were 100%, and *bla*_TEM_ gene was 94.44%.

**Table 2:** Sensitivity, specificity, and predictive value of multiplex RPA and tHDA assays.

Target genes	PCR	multiplex RPA		tHDA		Sensitivity%	Specificity%	PPV%	NPV%
		+	−	+	−				
*bla*_CTX-M_	+	95	0			100	100	100	100
*bla*_CTX-M_	−	0	49						
*bla*_OXA_	+	29	0			100	100	100	100
*bla*_OXA_	−	0	115						
*bla*_SHV_	+	2	0			100	100	100	100
*bla*_SHV_	−	0	142						
*bla*_TEM_	+			53	8	86.89	100	100	91.21
*bla*_TEM_	−			0	83				

+Positive; −Negative; *PPV* positive predictive value, *NPV* negative predictive value.

### Distribution of VF genes, phylogroups among ESBL variants

In the 95 ESBL-ExPEC clinical isolates, the VF genes exhibited the following order in frequency: *tra*T (67.4%) > *omp*T (52.6%) > *iut*A (50.5%) > *fim*H (47.4%) > *ih*a (33.7%) > *hly*A (26.3%) > *pap*C (12.6%) > *cva*C (3.2%), respectively (Table 3). All VF genes were distributed among CTX-M variants, excepting *pap*C and *cva*C that were not found in the CTX-M-27 variant. The *tra*T gene was found frequently in CTX-M-15 (75%), CTX-M-14 (64.3%), and CTX-M-55 (76.5%). While *iha* gene was predominate in CTX-M-27 (70.6%). Most common phylogroups among ESBL-ExPEC strains included: A (28.4%), B2 (28.4%), F (22.1%). The CTX-M-14, 15, and 55 (35.7%, 29.2%, and 47.1%) were predominant in phylogroup A, while most of CTX-M-27 belonged to phylogroup B2 (70.6%). Only CTX-M-15, and CTX-M-55 variants were found in the rare phylogroups B1, and E, respectively.

**Table 3:** Distribution of VF genes and phylogenetic groups in 95 CTX-M isolates.

CTX-M variants (Isolates containing)	Number of VF genes (%)								
	*tra*T	*omp*T	*iut*A	*fim*H	*iha*	*hly*A	*pap*C	*cva*C	Phylogroups (Number, %)
CTX-M-14 (14)	9 (64.3)	7 (50.0)	8 (57.1)	6 (35.71)	4 (28.6)	3 (21.4)	1 (7.1)	1 (7.1)	A (5, 35.7) B2 (3, 21.4) C (1, 7.1) D (2, 14.3) F (3, 21.4)
CTX-M-15 (48)	36 (75)	23 (47.9)	23 (47.9)	19 (39.6)	12 (25)	17 (35.4)	9 (18.8)	1 (2.1)	A (14, 29.2) B2 (9, 18) C (5, 10.4) D (7, 14.6) E (1, 2.1) F (12, 25)
CTX-M-27 (16)	6 (35.3)	11 (64.7)	9 (52.9)	11 (64.7)	12 (70.6)	2 (11.8)	0 (0)	0 (0)	B2 (12, 70.6) D (1, 5.9) F (3, 17.6),
CTX-M-55 (17)	13 (76.5)	9 (52.9)	8 (47.1)	9 (52.9)	4 (23.5)	3 (5.88)	2 (11.8)	1 (5.9)	A (8, 47.1) B1 (1, 5.9) B2 (3, 17.6) C (1, 5.9) D (1, 5.9) F (3, 17.6)
Total (95)	64 (67.4)	50 (52.6)	48 (50.5)	45 (47.4)	32 (33.7)	25 (26.3)	12 (12.6)	3 (3.2)	A (27, 28.4) B1 (1, 1.1) B2 (27, 28.4) C (7, 7.4) D (11, 11.6) E (1, 1.1) F (21, 22.1)

### Relationships of VF genes, phylogenetic groups, and antibiotic resistance

ESBL-ExPEC and non-ESBL-ExPEC isolates predominantly carried *tra*T and *omp*T, respectively (Table 4). Surprisingly, *cvc*C was absent in our non-ESBL isolates. The association of *tra*T, *omp*T, and *hlyA* were significantly different (*P* < 0.05) between the ESBL-ExPEC and non-ESBL-ExPEC isolates. Phylogroup predominance was as follows: phylogroup B2 (35.4%) > A (23.6%) > F (19.4%). ESBL-ExPEC were predominated by phylogroups A and B2, while non-ESBL-ExPEC were predominated by phylogroup B2. Only phylogroup B2 was significantly different between ESBL and non-ESBL groups (*P* < 0.05). Phylogroup Clades I was absent in all clinical isolates. Analysis of variance by Friedman’s test revealed a significant difference in VF gene distribution (*P* = 0.000). Three VF genes (*hly*A, *iha*, and *omp*T) were distributed differently across phylogroups (Table 5). Pairwise analysis of phylogroup showed that *hly*A was associated with phylogroup A and *iha* was associated with phylogroups F, A, and B2 (*P* < 0.05). Whereas, *omp*T was associated with phylogroups B2 and F (*P* < 0.05).

**Table 4:** Association of VF genes and phylogroups between ESBLs and non-ESBL clinical isolates.

VF genes (Number, %)	ESBL (n = 95)	Non-ESBL (n = 49)	^*a*^*P*	Phylogroups (Number, %)	ESBL (n = 95)	Non-ESBL (n = 49)	^*a*^*P*
*tra*T (84, 58.3)	64	20	0.002	A (34, 23.6%)	27	7	0.059
*omp*T (87, 60.4)	50	37	0.008	B1 (2, 1.4%)	1	1	0.632
*iut*A (79, 54.9)	48	31	0.147	B2 (51, 35.4%)	27	24	0.015
*fim*H (69, 47.9)	45	24	0.855	C (11, 7.6%)	7	4	0.865
*iha* (56, 38.9)	32	24	0.075	D (16, 11.1%)	11	5	0.804
*hly*A (26, 18.1)	25	1	< 0.001	E (2, 1.4%)	1	1	0.632
*pap*C (23, 16)	12	11	0.129	F (28, 19.4%)	21	7	0.326
*cvc*C (3, 2.1)	3	0	0.210	Clade I (0, 0%)	0	0	1.000

^*a*^*P* < 0.05 by Mann–Whitney U test.

**Table 5:** Relationship of VF genes and phylogroups in *E. coli* clinical isolates.

phylogroups	*tra*T	*omp*T	*iut*A	*fim*H	*iha*	*hly*A	*pap*C	*cvc*C
A	24	14	19	14	6	14	3	1
B1	1	1	2	1	1	0	0	0
B2	8	43	32	28	34	9	10	1
C	12	2	7	6	2	0	0	0
D	10	7	3	11	8	2	5	1
E	1	0	1	2	0	0	0	0
F	17	19	15	7	4	1	4	0
Clade I	0	0	0	0	0	0	0	0
^*a*^*P*	0.083	< 0.001	0.066	0.055	< 0.001	0.006	0.078	0.934

^*a*^*P* < 0.05 by Kruskal–Wallis test.

## Discussion

ExPEC is a major cause of morbidity and mortality in both hospital and community-acquired infections. Apart from epidemiological factors, acquisition of VFs and AMR, is likely to contribute to the global pandemic of ExPEC lineages^4,26^. Moreover, plasmid-mediated horizontal transfer of ESBL genes occur easily among species, causing widespread infection^6^. Accumulating evidence suggested that VFs helps gastrointestinal pathogens outcompete the commensal microbiota and impair host immunity via inducing colonization, resistance, and invasion^27^.

ESBL-*Enterobacteriaceae,* especially *E. coli* was one of the most frequent isolates in blood stream infection samples collected from a pediatric oncology center^28^. The MDR *E. coli* from febrile neutropenic cancer patients showed high resistance to ampicillin, cefepime, ceftriaxone, and cephradine^29^. Here, all ESBL-ExPEC isolated from cancer patients carried *bla*_CTX-M,_ showing the following predominance: *bla*_CTX-M1_ group (68.4%; 50.5% *bla*_CTX-M-15_ and 17.9% *bla*_CTX-M-55_) > *bla*_CTX-M9_ group (31.5%; 16.8% *bla*_CTX-M-27_ and 14.7% *bla*_CTX-M-14_). MDR was observed in all CTX-M variants. They were resistant to ceftriaxone, cefepime, ciprofloxacin, ceftazidime, levofloxacin, and gentamycin. Similarly, ESBL-ExPECs (from tertiary hospitals in Thailand) predominantly carried *bla*_CTX-M1_ (71.23%) and *bla*_CTX-M9_ (38.95%)^30^. The global pathogenic *E. coli* ST 131 strain harbors *bla*_CTX-M-15_ (67.6%), *bla*_CTX-M-27_ (20.6%), and *bla*_CTX-M-14_ (11.8%)^31^. Globally, the *bla*_CTX-M-15_ is frequently reported ESBL gene, especially in the bloodstream and urinary tract infections^23,32–34^. The *bla*_CTX-M-55_ is present in most *E. coli* isolated from pork and fecal samples^14,35^.

Several assays for genotyping ESBL genes, exist: (1) Kanokudom et al.,^14^ developed a naked-eye rapid multiplex RPA assay for detecting *bla*_CTX-M_, *bla*_OXA_, and *bla*_SHV_ in pork *E. coli* isolates. Amplicons were visualized on a single-stranded tag hybridization chromatographic printed-array strip (STH-PAS, a commercial lateral flow assay strip); (2) Higgins et al.,^36^ set up a portable loop-primer endonuclease cleavage-loop-mediated isothermal amplification (loop-primer endonuclease cleavage-LAMP) assay for detecting *bla*_CTX-M-1_ and *bla*_CTX-M-15_ in porcine fecal *E. coli* isolates; and (3) Wang et al. developed a probe-based real-time PCR assay for detecting *bla*_CTX-M_, *bla*_TEM_, and *bla*_SHV_ in broiler chicken *E. coli* isolates^37^^.^

Here, we developed the following isothermal assays to detect common ESBL genes: (1) a multiplex RPA assay to detect *bla*_CTX-M_, *bla*_OXA_, and *bla*_SHV_ genes; and (2) a tHDA assay to detect *bla*_TEM_ gene. The amplicons were visualized by agarose gel electrophoresis method, which is a cheap and widely used technique in general molecular laboratory. These simple isothermal platforms for nucleic acid amplification utilize only one pair of primers (like PCR) and the commonly available heating instruments. Moreover, compared to other isothermal platforms, the RPA kit (TwistAmp Basic kit) is a highly stable lyophilized reagent with long shelf life^38^. The lyophilized pellets in RPA kit are stable for at least one year when stored at temperatures below − 15 °C or at 2–8 °C, and up to 6 months at room temperature (22–28 °C)^39^. However, the RPA kit is slightly more expensive (~ $4.3 USD) than other amplification assays, such as PCR and LAMP (expired patents)^38^. Further, RPA patent is set to expire in 2023^40^, making way for the development of a new cost-effective in-house RPA formula which will allow for its large scale applications.

The nucleotide compositions and length of primers affect the optimum annealing temperature and incubation time in multiplex RPA reactions. Here high temperatures (leading to poor primers binding) or short incubation times did not yield *bla*_SHV_ amplicons, probably because the *bla*_SHV_ primers have lower GC contents and shorter lengths than *bla*_CTX-M_ and *bla*_OXA_ primers. High primer concentrations increases the chance of primer-dimer and non-specific amplicon formation in multiplex RPA reactions, while low concentrations may lead to low yields. Therefore, good primer designing and extensive optimization are critical for effective multiplexed isothermal amplification^41^. The LOD of our multiplex RPA-gel electrophoresis assay in detecting *bla*_CTX-M_, *bla*_OXA_, and *bla*_SHV_ genes was slightly lower than that of the previous multiplex RPA-Lateral Flow Assay (LFA)^14^. RPA amplicons must be purified before loading onto the agarose gel to remove protein contaminants^42^. This RPA post-amplification purification step causes amplicon loss. Moreover, the detection sensitivity using agarose gel electrophoresis depends on efficacy of various steps (pre-loading, pre-casting, and post-staining)^43^. Thus, the RPA post-amplification purification is a major drawback for gel electrophoresis detection^44^. Several methods for RPA products purification exist, including heat denaturation (65 °C or 95 °C for 10 min), sodium dodecyl sulfate treatment, proteinase K digestion, protein sedimentation via high-speed centrifugation and purification using commercial DNA purification kits^44^. Although agarose gel electrophoresis is a common method for visualization of amplification products, it is time consuming due to gel preparation, electrophoresis, gel staining, and imaging steps. These limitations can be circumvented in the future—by using various in-house detection methods (which employ bridge flocculation, SYBR green I or lateral flow assays)—to develop a cost-effective, simple, equipment-free, rapid, and naked-eye assay for detecting the desired genes^39,44^.

Assessing LOD of tHDA assay for *bla*_TEM_ required 10x–100 × the template concentration used in RPA assays for *bla*_CTX-M_, *bla*_OXA_, *bla*_SHV_ genes. The high-temperature tHDA operating condition (60–65 °C) may affect the primer binding efficiency; however specificity could be higher than mesophilic isothermal amplification^38^. Moreover, compared to RPA and PCR, we used lower primer concentrations (0.1–1 μM vs. 75–100 nM) for tHDA assay. The tHDA amplicon is generally < 150 nucleotides long, due to helicase processivity which limits the multiplexing capacity^41,45^. Our multiplex RPA assay showed 100% sensitivity and specificity for *bla*_CTX-M_, *bla*_OXA_, and *bla*_SHV_ genes. However, tHDA had 86.89% sensitivity and 100% specificity for *bla*_TEM_. The higher LOD and lower primer concentrations used may have caused tHDA assay to have lower sensitivity, as compared to the RPA assay. The false-negative results may occur for samples with low quantity and quality of DNA (old/degraded DNA).

ExPEC strains, compared to commensal *E. coli* strains, have complex phylogenetic structure and diverse VFs^4^. ExPEC strains, such as uropathogenic *E. coli* (UPEC), neonatal meningitis *E. coli* (NMEC), sepsis-associated *E. coli* (SEPEC), and avian pathogenic *E. coli* (APEC), have several VFs. ExPEC VFs—such as adhesins, toxins and iron acquisition, lipopolysaccharides, capsules, and invasins—facilitate their colonization and systemic infection. UPEC prevalently causes urinary tract infection and secondary bacteremia^18^. Here, three VF genes (*tra*T, *omp*T, and *hly*A) were significantly associated with ESBL-ExPEC strains. *tra*T was the most prevalent one, present in CTX-M-14, 15, and 27 variants. Other prevalent VF genes were aerobactin acquisition (*iut*A) and adhesins (*fim*H and *iha*). While a few *cva*C existed only in our ESBL-ExPEC clinical isolates. The serum resistance gene *tra*T, which inhibits the classical pathway of complement activity, is present in all pathotypes with more prevalence in UPEC^46–48^. The outer membrane protein (*omp*T) associated with UPEC enables intracellular survival and evasion from the host defense. Hemolysin A (*hly*A) is a membrane lysis toxin present in UPEC strains. The afimbrial adhesin (*afa*) was found associated with bacteremia mortality^23^.

Only phylogroup B2 was significantly different between ESBL-ExPEC and non-ESBL-ExPEC. Similarly, the extended-spectrum cephalosporin resistant *E. coli* isolates belonging to phylogroup B2 carried ESBL and/or plasmid-mediated AmpC genes; *bla*_CTX-M-15_, *bla*_CTX-M-14_, *bla*_CTX-M-55_ were the most prevalent^49^. The phylogroup A and F are commensal and avian associated strains^50^, respectively. They are the second and third abundant phylogroups in our *E. coli* isolates. In addition, only three VF genes (*omp*T, *iha*, and *hly*A) were distributed differently across phylogroups, especially in B2, F, and A. The phylogroup B2 was the most prevalent in clinical isolates (from many systemic infections) and carried a greater number of VFs than other phylogroups^4^. Although phylogroup F was less virulent than B2, its clinical significance in mediating AMR in ExPEC is established^49,51,52^.

In conclusion, most ESBL-ExPECs possessed a MDR pattern belonging to phylogroup B2 and carried more VF genes. The *tra*T, *omp*T, and *hly*A were found associated with ESBL-ExPEC strains. The relationship of VF genes was first demonstrated across phylogroups with diverse *omp*T, *iha*, and *hly*A. The *E. coli* pathotypes should be characterized in the future. The identification and characterization of AMR, VF genes, and their pathotypes and phylogenetic analysis may contribute to developing novel strategies for treating *E. coli*-mediated systemic infection. Moreover, our multiplex RPA and tHDA assays, presented here, are simple, rapid, and reliable in detecting the most common ESBLs genes. These assays are beneficial for strategizing targeted therapy in low resource settings (using common heating equipments) and epidemiological control.

## Materials and methods

### Bacterial isolates

A total of 144 *E. coli* clinical isolates from various specimens including blood, urine, pus, body fluid, and sputum were collected from a tertiary cancer institute in Bangkok (Thailand) between February 2017–September 2018. All isolates were preserved in skimmed milk at − 80 °C until use.

The sample size used here was calculated using the Buderer method,^53^ which helps evaluate sensitivity and specificity of diagnostic tests at a 95% confidence interval. The study was approved by the Ethical Committee of the National Cancer Institute, Thailand (certificate number 020/2562).

### Antimicrobial susceptibility testing and ESBLs detection

Ninety-five ESBL-ExPEC and forty-nine non-ESBL-ExPEC isolates were selected consecutively and non-duplicate from clinical samples by phenotypic ESBLs screening. Their antimicrobial susceptibility to various antibiotics—including, amikacin (30 µg), gentamicin (10 µg), ampicillin (10 µg), amoxicillin/clavulanic acid (20/10 µg), ceftazidime (30 µg), cefotaxime (30 µg), cefdinir (5 µg), cefepime (30 µg), ciprofloxacin (5 µg), ceftriaxone (30 µg), doripenem (10 µg), ertapenem (10 µg), imipenem (10 µg), meropenem (10 µg), levofloxacin (5 µg), and piperacillin/tazobactam (1.25/23.75 µg)—was determined using disk diffusion method. The ESBLs confirmatory testing was carried out using the combination disk method according to the CLSI guidelines^11^. ESBLs was considered to be produced when size of either inhibition zones of cefotaxime/clavulanic acid (30/10 µg) or ceftazidime/clavulanic acid (30/10 µg) were ≥ 5 mm compared to that of cefotaxime (30 µg), or ceftazidime (30 µg).

### DNA extraction

DNA was isolated from *E. coli* pure colonies cultured on MacConkey agar by boiling method. Briefly, bacterial isolates were suspended in 400 µL of Tris–EDTA buffer (TE buffer), vortex mixed, and boiled at 95°C for 10 min. The supernatant was separated by centrifugation at 12,000 rpm for 5 min. DNA was precipitated by adding 1/10 volume of 3 M NaOAc, pH 5.2, and 2–3 volumes of chilled absolute ethanol and centrifuged at 12,000 rpm for 15 min. The bacterial DNA was washed with 70% ethanol, dried, and dissolved in 50 µL of TE buffer. DNA was stored at − 20 °C for subsequent amplification.

### PCR amplification and DNA Sequencing

All *E. coli* clinical isolates were investigated for the presence of ESBL genes (*bla*_CTX-M_, *bla*_OXA_, *bla*_SHV_, and *bla*_TEM_). Further, the genes were sequenced to identify the variants. The primer sequences and amplicon sizes of each ESBL gene are described in Supplementary Table S2. The PCR reaction was carried out in a total volume of 25 µL comprising 50 ng of DNA, 1.5 mM MgCl_2_, 0.2 mM dNTPs, 0.4 µM of each primer, 1 × Standard *Taq* reaction buffer, and 1.25 U *Taq* polymerase (New England Biolabs, UK). The amplification of each ESBL gene were performed as previously described^54–57^. Positive control of *bla*_TEM_, *bla*_CTX-M,_ *bla*_OXA_, and *bla*_SHV_ were derived from *E. coli* EC137 and *K.pneumoniae* KP125, respectively (kindly provided by Prof. Visanu Thamlikitkul, Faculty of Medicine Siriraj Hospital, Mahidol University, Bangkok, Thailand). All PCR products were examined using 2% agarose gel electrophoresis. The amplified fragments were sequenced (Bioneer Corporation, South Korea) and aligned with the GenBank database using the BLASTn program (https://blast.ncbi.nlm.nih.gov/) and Clustal Omega (https://www.ebi.ac.uk/Tools/msa/ ustalo/), respectively.

### Multiplex RPA reaction for ***bla***_CTX-M_, ***bla***_OXA_, and ***bla***_SHV_ genes

The RPA primers for *bla*_CTX-M_, *bla*_OXA_, and *bla*_SHV_, genes described previously were used (Supplementary Table S3). The multiplex RPA reaction was performed using the TwistAmp Basic reaction kit (TwistDx, UK). The RPA master mix contained 0.2 µM of *bla*_CTX-M_ primers, 0.1 µM each of *bla*_OXA_ and *bla*_SHV_ primers, 29.5 µL rehydration buffer, 50 ng of DNA template, and sterile distilled water added to obtain a final volume of 47.5 µL. The reaction was vortex mixed, and then transferred to a freeze-dried tube, and finally mixed with 2.5 µL of 280 mM MgOAc. The reaction was incubated at 37 °C for 25 min. To explore the optimum conditions of the multiplex RPA reactions for *bla*_CTX-M_, *bla*_OXA_, and *bla*_SHV_, the following conditions were tried: (a) two sets of CTX-M, OXA, and SHV primer concentrations—0.2, 0.1, 0.1 and 0.2, 0.05, 0.05 µM; (b) three reaction temperatures—37 °C, 39 °C, and 41 °C; and (c) five incubation durations—10, 15, 20, 25, and 30 min. The RPA amplicons were purified using a GeneJet PCR purification kit (Thermo Fisher Scientific Inc., USA) and detected using 2% agarose gel electrophoresis.

### tHDA reaction for ***bla***_TEM_ gene

The *bla*_TEM_ gene of *E. coli* (accession no. MG515250.1) was used for primer design using Primer3plus program (http://www.bioinformatics.nl/cgi-bin/primer3plus/primer3plus.cgi). The sequences were as follows: forward primer, 5′-TGAGTGATAACACTGCGGCCAACTTAC-3′; reverse primer, 5′-CCCTACGATCAAGGCGAGTTACATGAT-3′. The tHDA reaction was performed in a total volume of 50 µL using IsoAmp® II Universal tHDA Kit (New England Biolabs, Inc., USA). The reaction mixer contained 1X Annealing buffer II, 4 mM MgSO4, 40 mM NaCl, 3.5 µL IsoAmp® dNTP Solution, 0.075 µM of each primer, and 3.5 µL IsoAmp® Enzyme Mix. The reaction was overlaid with mineral oil and incubated at 67 °C for 75 min. The tHDA condition was optimized by varying primer concentration (0.025, 0.050, and 0.075 µM), temperature (63 °C, 65 °C, 67 °C, 69 °C, 71 °C) and incubation time (30, 45, 60, 75, and 90 min). The tHDA amplicon of 111 bp was detected using 2% agarose gel electrophoresis.

### Analytical LOD and specificity of multiplex RPA and tHDA assays

The template DNA from *E. coli* EC120, *K. pneumoniae* KP125, *K. pneumoniae* ATCC 700,603, *E. coli* EC137 harboring *bla*_CTX-M,_ *bla*_OXA_, *bla*_SHV_, and *bla*_TEM_ genes, respectively, were diluted to 100, 50, 5, 0.5, 0.05 ng/µL concentrations. The LOD was assessed as the lowest DNA concentration required for amplicon production using the optimized (in this study) multiplex RPA and tHDA assays. The specificity was also evaluated using DNA isolated from *E. coli* ATCC 25,922, *K.* pneumoniae ATCC 700,603, *Proteus mirabilis* ATCC 25,933, *Pseudomonas aeruginosa* ATCC 27,853, *Acinetobacter baumannii* ATCC 19,606, *Staphylococcus aureus* ATCC 25,923, and *Enterococcus faecalis* ATCC 29,212.

### Validation of multiplex RPA and tHDA assays

All 144 clinical isolates were examined for the presence of *bla*_CTX-M_, *bla*_OXA_, and *bla*_SHV_ genes (multiplex RPA assay) and *bla*_TEM_ gene (tHDA assay). The sensitivity, specificity, positive and negative predictive values were calculated by comparing with nucleotide sequencing results obtained using MEDCALC® easy-to-use statistical software (https://www.medcalc.org/calc/diagnostic_test.php).

### Detection of virulence genes

Eight virulence genes, viz. *tra*T, *omp*T, *iut*A, *fim*H, *hly*A, *iha*, *pap*C, and *cva*C, which are commonly associated with ExPEC were characterized by multiplex PCR methods. Three positive control genes (*fim*H, *hly*A, and *iut*A) were synthesized and commercially cloned in GeneArt vectors (Invitrogen, USA). The recombinant plasmids were then transformed into a DH5α competent cell using Subcloning Efficiency™ DH5α Competent Cell (Invitrogen, USA). The primers for these three virulence genes (designed here) and five primers sets for *tra*T, *omp*T*, cva*C, *iha*, and *pap*C genes (described previously) are tabulated in Supplementary Table S4. The first pool consists of *fim*H, *hly*A, and *iut*A*,* while the second pool comprises *cva*C, *iha*, *tra*T, *omp*T, and *pap*C. A total 25 µL reaction comprising 1 × Standard *Taq* reaction buffer and 1.5 U *Taq* polymerase (New England Biolabs, UK), 0.2 µM of each set of primers (forward and reverse), 0.2 mM dNTPs, ~ 100 ng DNA template and ultra-pure water were used. The optimized PCR condition for virulence genes was: initial denaturation at 94 ℃ for 15 min followed by 29 cycles at 94 ℃ for 1 min, annealing at 56 ℃ for 1 min, extension at 72 ℃ for 1 min, and post extension at 72 ℃ for 10 min. All amplicons were analyzed using 2% agarose gel electrophoresis.

### Phylogroup analysis

Quadruplex PCR was performed by targeting *arp*A, *chu*A, *yja*A, and *TspE4.*C2 (Clermont et al.). An additional PCR was carried out as described previously^20^ using specific primers for groups C, E, and cryptic Clades. Previously published primers and the amplicon size are illustrated (Supplementary Table S5). The phylogroups B1, B2, and F were assigned based on quadruplex results, while specific primers were used to differentiate phylogroups C, E, and other cryptic Clades from A, D, and Clade I^20^. A total reaction of 25 µL consisting of 2.5 µL 10 × buffer, 0.2 mM of each dNTPs, 1 µL DNA template (100 ng), 1.5 U Taq polymerase (New England Biolabs, UK), 0.2 µM primers except for internal control (*trp*BA = 0.12 µM) and Milli Q water was used. We followed the same PCR conditions as described by Clermont et al. 2013^20^. PCR products were analyzed using 2% agarose gel electrophoresis.

### Statistical analysis

The association of VF genes and phylogroups was compared between ESBL and non-ESBL-ExPEC isolates using Mann–Whitney U test. The distribution of VF genes across phylogroups was tested using Friedman’s and Kruskal–Wallis tests. *P* value < 0.05 was considered statistically significant.

### Ethical approval

The study protocol was approved by Research Committee of National Cancer Institute (certificate number 020/2562).

## Supplementary Information

Supplementary Tables.

## Data Availability

The datasets generated during the current study are available in the genbank NCBI repository, with the accession number including OP999005-OP999011. These datasets were derived from the following public domain resources: https://www.ncbi.nlm.nih.gov/nuccore/OP999005, https://www.ncbi.nlm.nih.gov/nuccore/OP999006, https://www.ncbi.nlm.nih.gov/nuccore/OP999007, https://www.ncbi.nlm.nih.gov/nuccore/OP999008, https://www.ncbi.nlm.nih.gov/nuccore/OP999009, https://www.ncbi.nlm.nih.gov/nuccore/OP999010, https://www.ncbi.nlm.nih.gov/nuccore/OP999011.
