# Supplementary material for: Detection of Extended-spectrum β-lactamase-producing Escherichia coli isolates by isothermal amplification and association of their virulence genes and phylogroups with extraintestinal infection
Source: Sci Rep. 2023 Jul 25;13:12022. doi: 10.1038/s41598-023-39228-w (PMC10368679; doi:10.1038/s41598-023-39228-w)
Supplement: Supplementary file 1 — Supplementary Tables. [file 41598_2023_39228_MOESM1_ESM.pdf]

Detection of Extended spectrum β-lactamase-producing *Escherichia coli* isolates by isothermal amplification and their virulence genes and phylogroup association with extraintestinal infection

Naeem Ullah[1], Thadchaporn Assawakongkarat[2], Yukihiro Akeda[3,4], *Nuntaree* Chaichanawongsaroj[1*]

Short title: RPA and tHDA for *bla*$_{CTX-M}$, *bla*$_{OXA}$, *bla*$_{SHV}$, *bla*$_{TEM}$ genes

[1]Research Unit of Innovative Diagnosis of Antimicrobial Resistance, Department of Transfusion Medicine and Clinical Microbiology, Faculty of Allied Health Sciences, Chulalongkorn University, Bangkok, Thailand.

[2]Program of Molecular Sciences in Medical Microbiology and Immunology, Department of Transfusion Medicine and Clinical Microbiology, Faculty of Allied Health Sciences, Chulalongkorn University, Thailand.

[3]Division of Infection Control and Prevention, Osaka University Hospital, Osaka University.

[4]Research Institute for Microbial Diseases, Osaka University, 565-0871, Japan.

[*]Corresponding author

E-mail: nuntaree@gmail.com (NC)

Table S1. Molecular characterization of *bla* genes among clinical ESBL-producing *E. coli* isolates (n = 95)

**Genotype of *bla* gene**	**ESBLs producing *E. coli*** **N = 95 (Percentage)**
CTX-M-14	7 (7.4)
CTX-M-14, TEM-1	4 (4.2)
CTX-M-14, TEM-1, OXA-1	2 (2.1)

Genotype of *bla* gene	ESBLs producing *E. coli*<br>N = 95 (Percentage)
CTX-M-14, TEM-1, OXA-1, SHV	1 (1.1)
CTX-M-15	16 (16.8)
CTX-M-15, OXA-1	13 (13.6)
CTX-M-15, TEM-1	7 (7.4)
CTX-M-15, TEM-1, OXA-1	11 (11.6)
CTX-M-15, TEM-1, SHV	1 (1.1)
CTX-M-27	13 (13.7)
CTX-M-27, OXA-1	1 (1.1)
CTX-M-27, TEM-1	2 (2.1)
CTX-M-55	5 (5.3)
CTX-M-55, TEM-1	11 (11.6)
CTX-M-55, TEM-1, OXA-1	1 (1.1)

Table S2. Primer sequences and PCR product sizes of ESBLs genes

Gene	Primer name	Sequence 5' to 3'	Product Size (bp)	Ref
*bla*TEM	TEM-F	TCCGCTCATGAGACAATAACC	931	1
	TEM-R	TTGGTCTGACAGTTACCAATGC		
*bla*CTX-M	CTX-M-F	ATGTGCAGYACCAGTAARGTKATGGC	593	2
	CTX-M-R	TGGGTRAARTARGTSACCAGAAYCAGCGG		
*bla*OXA	OXA-F	ACACAATACATATCAACTTCGC	813	3

	OXA-R	AGTGTGTGTTTAGAATGGTGATC		
bla$_{SHV}$	SHV-F	TGGTTATGCGTTATATTCGCC	870	[4]
	SHV-R	GGTTAGCGTTGCCAGTGCT		

Table S3. RPA primer sequences and product sizes for ESBLs genes

Gene	Primer	Sequence 5' to 3'	Product size (bp)	Ref
bla$_{CTX-M}$	CTX-M-F	ATGTGCAGYACCAGTAARGTKATGGC	593	[2]
	CTX-M-R	TGGGTRAARTARGTSACCAGAAYCAGCGG		
bla$_{OXA}$	OXA-F	ATTATCTACAGCAGCGCCAGTG	296	[5]
	OXA-R	TGCATCCACGTCTTTGGTG		
bla$_{SHV}$	SHV-F	GATGAACGCTTTCCCATGATG	214	
	SHV-R	CGCTGTTATCGCTCATGGTAA		

Table S4. Primer sequences for VF genes identification and their product sizes

Gene	Primer name	Sequence 5' to 3'	Product Size (bp)	Ref
*fim*H	fimH-F	TCCCTACTACCAGCGAAAC	482	TS
	fimH-R	CCCTACTGCTCCTAACGATAC		
*hly*A	hlyA-F	CCAGAAGCAAGTCTTTGACC	309	TS
	hlyA-R	ACCTTATCATCCCCGTCTCCCAGG		
*iut*A	iutA-F	CTATAAAAGCCAGGGCGAC	277	TS
	iutA-R	CTGCTGTGACGAAGAGAAAG		

*cva*C	cvaC-F	CACACACAAACGGGAGCTGTT	680	[6]
	cvaC-R	CTTCCCGCAGCATAGTTCCAT		
iha	iha-F	AACTGGCAGATCACCGAAGA	346	[7]
	iha-R	GCGACATCCAGTAATTTCGCT		
*tra*T	traT-F	GGTGTGGTGCGATGAGCACAG	290	[6]
	traT-R	CACGGTTCAGCCATCCCTGAG		
*pap*C	papC-F	GTGGCAGTATGAGTAATGACCGTTA	200	[6]
	papC-R	ATATCCTTTCTGCAGGGATGCAATA		
*opm*T	ompT-F	TTTGATGCCCCAGATATCTATCGG	236	[8]
	ompT-R	GGCTTTCCTGATATCCGGCCATG		

Table S5. Primer sequences and amplicon sizes used for phylogenetic analysis

Gene	Primer name	Sequence 5' to 3'	Product Size (bp)	Ref
*chu*A	chuA-F	ATGGTACCGGACGAACCAAC	288	[9]
	chuA-R	TGCCGCCAGTACCAAAGACA		[10]
*yja*A	yjaA-F	CAAACGTGAAGTGTCAGGAG	211	[9]
	yjaA-R	AATGCGTTCCTCAACCTGTG		
TspE4.C2	TspE4.C2-F	CACTATTCGTAAGGTCATCC	152	
	TspE4.C2-R	AGTTTATCGCTGCGGGTCGC		
*arp*A	Acek-F	AACGCTATTCGCCAGCTTGC	400	

	arpA1-F	TCTCCCCATACCGTACGCTA		[11]
*trp*A	trpAgpE-F	AGTTTTATGCCCAGTGCGAG	219	[12]
	trpAgpE-R	TCTGCGCCGGTCACGCCC		
*arp*A	ArpAgpE-F	GATTCCATCTTGTCAAAATATGCC	301	
	ArpAgpE-R	GAAAAGAAAAAGAATTCCCAAGAG		
*trp*A	trpBA-F	CGGCGATAAAGACATCTTCAC	489	[13]
	trpBA-R	GCAACGCGGCCTGGCGGAAG		

References

1. Stürenburg, E., Lang, M., Horstkotte, M. A., Laufs, R. & Mack, D. Evaluation of the MicroScan ESBL plus confirmation panel for detection of extended-spectrum β-lactamases in clinical isolates of oxyimino-cephalosporin-resistant Gram-negative bacteria. *J. Antimicrob. Chemother.* **54**, 870–875; 10.1093/jac/dkh449 (2004).
2. Hasman, H., Mevius, D., Veldman, K., Olesen, I. & Aarestrup, F. M. β-Lactamases among extended-spectrum β-lactamase (ESBL)-resistant *Salmonella* from poultry, poultry products and human patients in The Netherlands. *J. Antimicrob. Chemother.* **56**, 115–121; 10.1093/jac/dki190 (2005).
3. Costa, D. *et al.* Detection of *Escherichia coli* harbouring extended-spectrum β-lactamases of the CTX-M, TEM and SHV classes in faecal samples of wild animals in Portugal. *J. Antimicrob. Chemother.* **58**, 1311–1312; 10.1093/jac/dkl415 (2006).
4. Kim, J., Kwon, Y., Pai, H., Kim, J.-W. & Cho, D.-T. Survey of *Klebsiella pneumoniae* strains producing extended-spectrum β-lactamases: prevalence of SHV-12 and SHV-2a in Korea. *J. Clin. Microbiol.* **36**, 1446–1449; 10.1128/JCM.36.5.1446-1449.1998 (1998).
5. Kim, J. *et al.* Rapid detection of extended spectrum β-lactamase (ESBL) for *Enterobacteriaceae* by use of a multiplex PCR-based method. *Infect. Chemother.* **41**, 181–184; 10.3947/ic.2009.41.3.181 (2009).

6. Johnson, J. R. & Stell, A. L. Extended virulence genotypes of *Escherichia coli* strains from patients with urosepsis in relation to phylogeny and host compromise. *J. Infect. Dis.* **181**, 261–272; 10.1086/315217 (2000).
7. Colello, R. *et al.* Identification and detection of *iha* subtypes in LEE-negative *Shiga toxin*-producing *Escherichia coli* (STEC) strains isolated from humans, cattle and food. *Heliyon* **5**, e03015; 10.1016/j.heliyon.2019.e03015 (2019).
8. Desloges, I. *et al.* Identification and characterization of OmpT-like proteases in uropathogenic *Escherichia coli* clinical isolates. *Microbiologyopen* **8**, e915; 10.1002/mbo3.915 (2019).
9. Clermont, O., Christenson, J. K., Denamur, E. & Gordon, D. M. The Clermont *Escherichia coli* phylo-typing method revisited: improvement of specificity and detection of new phylo-groups. *Environ. Microbiol. Rep.* **5**, 58–65; 10.1111/1758-2229.12019 (2013).
10. Clermont, O., Bonacorsi, S. & Bingen, E. Rapid and simple determination of the *Escherichia coli* phylogenetic group. *Appl. Environ. Microbiol.* **66**, 4555–4558; 10.1128/AEM.66.10.4555-4558.2000 (2000).
11. Clermont, O., Bonacorsi, S. & Bingen, E. Characterization of an anonymous molecular marker strongly linked to *Escherichia coli* strains causing neonatal meningitis. *J. Clin. Microbiol.* **42**, 1770–1772; 10.1128/JCM.42.4.1770-1772.2004 (2004).
12. Lescat, M. *et al.* Commensal *Escherichia coli* strains in Guiana reveal a high genetic diversity with host-dependant population structure. *Environ. Microbiol. Rep.* **5**, 49–57; 10.1111/j.1758-2229.2012.00374.x (2013).
13. Clermont, O. *et al.* Evidence for a human-specific *Escherichia coli* clone. *Environ. Microbiol.* **10**, 1000–1006; 10.1111/j.1462-2920.2007.01520.x (2008).
